# Rh → Sb Interactions Supported by Tris(8-quinolyl)antimony Ligands

**DOI:** 10.1021/acs.organomet.4c00258

**Published:** 2024-08-15

**Authors:** Casey R. Wade, Brendan L. Murphy, Shantabh Bedajna, François P. Gabbaï

**Affiliations:** † Department of Chemistry & Biochemistry, The Ohio State University, Columbus, Ohio 43210, United States; ‡ Department of Chemistry, Texas A&M University, College Station, Texas 77843, United States

## Abstract

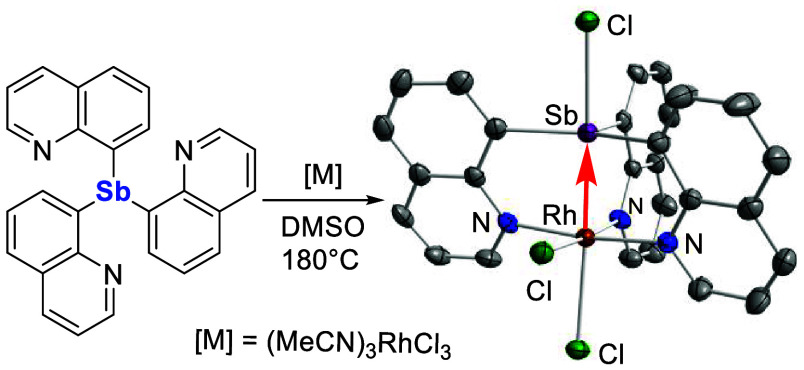

The ligands tris(8-quinolyl)stibine and tris(6-methyl-8-quinolyl)stibine have been synthesized and complexed to rhodium using (MeCN)_3_RhCl_3_. The resulting complexes feature an unusual [RhSb]^VI^ core as a result of the formal insertion of the antimony center into one of the Rh–Cl bonds. Computational analysis using density functional theory (DFT) methods reveals that the resulting Rh–Sb σ bond is polarized toward the Rh atom, suggesting a description of this linkage as a Rh → Sb Z-type interaction.

The study of ambiphilic systems combining L-type and Z-type ligands within the same construct has emerged as a field of active investigation, especially in the cases of ligands containing a group 13 element as a σ-acceptor for transition metals.^1^ Parallel to these developments, several groups have investigated more atypical systems in which the Z-type ligand is a group 15 element.^2^ Our contributions to this area have focused on the use of phosphinostibine ligands for the generation of transition metal complexes in which the antimony moiety acts as a Z-type ligand.^1d,3^ We have shown that the magnitude of the resulting M → Sb interaction can be readily modulated by the oxidation state of the antimony atom^4^ as well as its charge which can be manipulated by abstraction of anionic ligands.^6^ Our work has also shown that these effects can be leveraged to enhance the catalytic properties of the transition metal center.^4,6^ Some of the simplest systems that we have investigated are those resulting from the reaction of platinum dichloride with the bis- or tris-phosphinostibines ClSb(*o*-dppp)_2_ and Sb(*o*-dppp)_3_, respectively (*o*-dppp = *o*-(Ph_2_P)C_6_H_4_). These reactions proceed by oxidative insertion of the stibine into a Pt- Cl bond to produce complexes **A** and **B**,^7^ respectively (Chart 1). Reasoning that the properties of these complexes may also be influenced by the nature of the L-type buttresses, we have now questioned whether stibines featuring nitrogen donor ligands could also display the redox noninnocence of their phosphine counterparts and support the formation of such complexes. Following up on some of our work with ambiphilic tellurium-quinoline ligands,^8^ we now report on the reaction of tris-(8-quinolyl)stibines^50^ toward (MeCN)_3_RhCl_3_.

**Chart 1:**
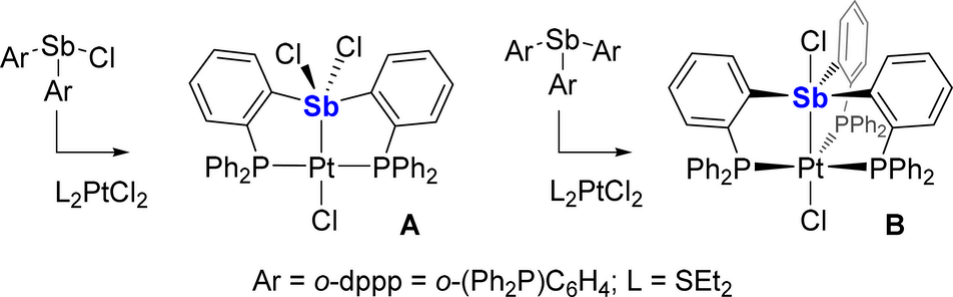
Oxidative Insertion of Antimony Centers of Phosphinostibines into Pt–Cl Bonds

The target ligand (**L**_**Quin**_) was accessed as a yellow powder in 74% yield by reacting 3 equiv of 8-lithioquinoline with one equiv of SbCl_3_ in THF at −78 °C (Figure 1). The ^1^H NMR spectrum displays the expected six resonances of a substituted quinolyl group (Figure S1). Single crystals allowed for the determination of the solid-state structure of the ligand (Figure 1), which reveals short contacts between the central Sb and the quinolyl N atoms (avg. 3.08 Å), suggesting three convergent secondary interactions^9^ sometimes referred to as pnictogen bonds.^10^ Further reaction of **L**_**Quin**_ with (MeCN)_3_RhCl_3_ in boiling DMSO (Figure 1) gave rise to a yellow crystalline powder (**1**) for which combustion analysis was consistent with the formation of a 1:1 complex between **L**_**Quin**_ and RhCl_3_ (see Supporting Information). Unfortunately, the poor solubility of **1** precluded its characterization by multinuclear NMR spectroscopy. Consequently, a more soluble version of such a complex was targeted by metalation of the newly prepared tris(6-methyl-8-quinolyl)stibine (**L**_**Quin-Me**_) with (MeCN)_3_RhCl_3_. The product of this reaction (**2**) was analyzed by ^1^H NMR spectroscopy, which shows two sets of quinolyl resonances in a 2:1 ratio, suggesting ligation of the three Lewis basic quinolyl moieties to the Rh(III) center (Figure S5).

**Figure 1:**
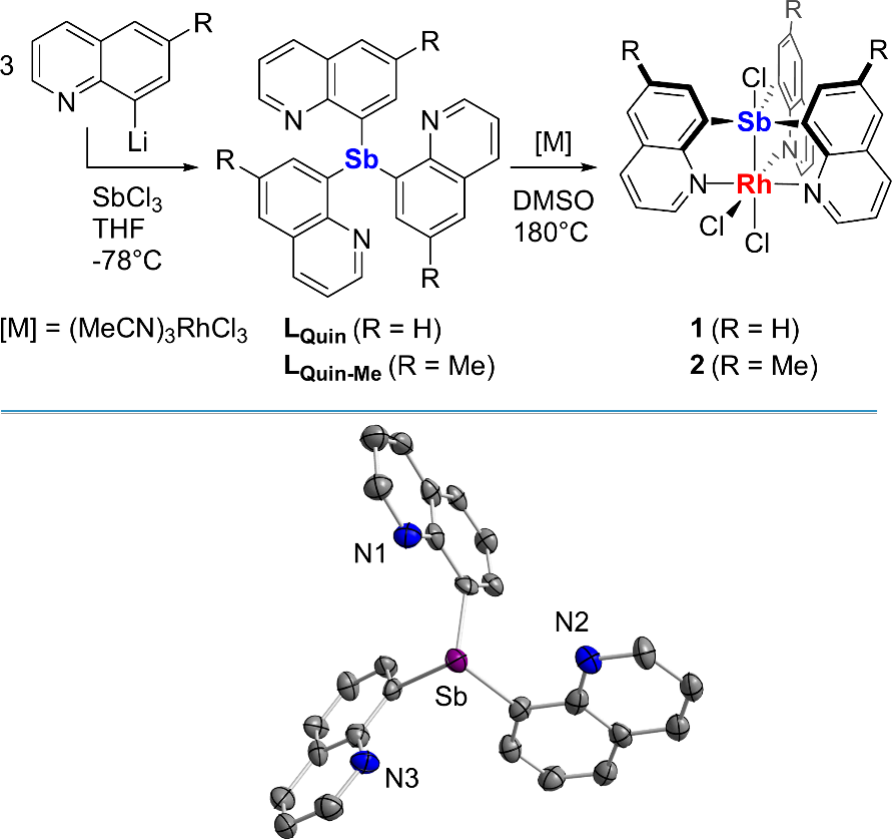
Top: Synthesis of **L**_**Quin**_, **L**_**Quin-Me**_, **1**, and **2**. Bottom: Solid-state structure of **L**_**Quin**_. Hydrogen atoms are omitted for clarity. Color code: purple (Sb), blue (N), gray (C).

The solid-state structure of **1** was determined using single crystals obtained from DMSO. Examination of this structure reveals the formal insertion of the central antimony atom into a Rh–Cl bond with the three quinolyl ligands occupying the remaining coordination sites of the pseudo-octahedral Rh(III) metal center (Figure 2). The resulting Sb–Cl3 bond is quite short (2.5414(18) Å) compared to the Sb–Cl bonds found in **B** (2.686(1) Å),^7b^ suggesting that the trivalent rhodium center in **1** is more electron-withdrawing than the divalent platinum center of **B**.^7b^ In support of this view, the Sb–Cl distance in **1** is quite similar to that found in Ph_3_SbCl_2_ (avg. 2.46 Å) which features two electron-withdrawing chloride ligands trans to one another.^11^ The rigid quinolyl backbone may be held responsible for the narrow Sb–Rh separation of 2.4662(13) Å, which is shorter than the Sb–Rh bonds found in *mer*-[RhCl_3_(SbPh_3_)_3_] (avg. 2.59 Å)^12^ but on par with those found in the tris-phosphinostibine-rhodium complex **C** (2.420(1) Å)^2a^ and the paddlewheel Rh/Sb heterobimetallic complex **D** (2.4910(2) Å) (Chart 2).^13^ Lastly, the central Sb atom is positioned trans to a chloride ligand, resulting in a Rh–Cl1 distance (2.7071(19) Å) that is substantially longer than that of the orthogonal Rh–Cl2 bond (2.3585(17) Å). The lengthening of the Rh–Cl1 bond is correlated to the electronic structure of the Sb–Rh bond, for which the electron density is polarized toward the rhodium center (*vide infra*). The solid-state structure of **2** was also determined using crystals obtained from CDCl_3_/*o*-difluorobenzene (see Figure S6). This complex features comparable structural characteristics to **1**, exhibiting Sb–Cl3, Sb–Rh, and Rh–Cl1 bond distances of 2.5242(19), 2.4684(7), and 2.740(2) Å, respectively.

**Figure 2:**
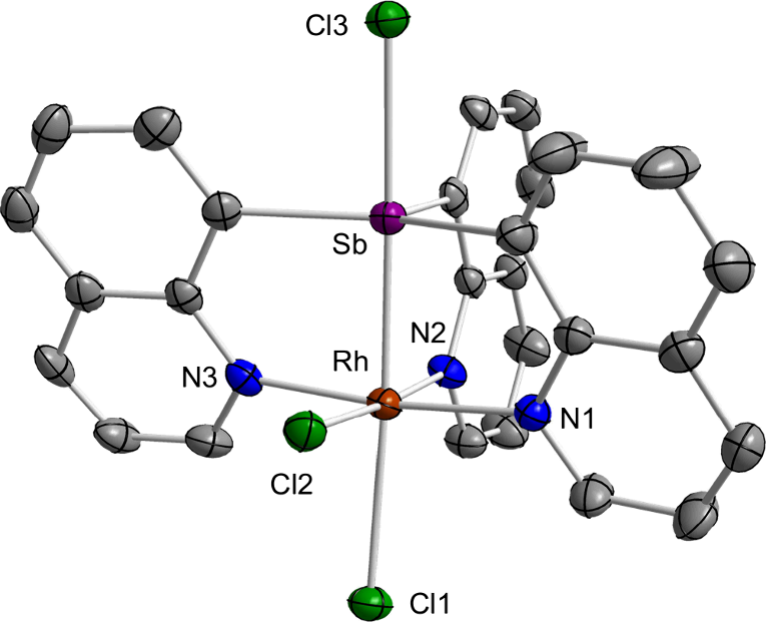
Solid-state structure of **1**. Thermal ellipsoids are drawn at the 50% probability level. Hydrogen atoms and interstitial DMSO molecules are omitted for clarity. Pertinent metrical parameters can be found in the text. Color code: purple (Sb), orange (Rh), green (Cl), blue (N), gray (C).

**Chart 2:**
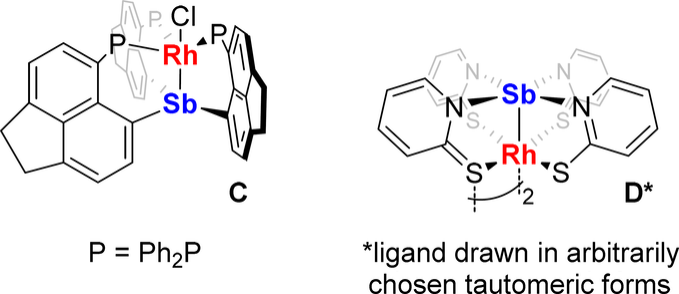
Related Rh/Sb Heterobimetallic Complexes

With these complexes in hand, we sought to understand the bonding between the Rh and Sb centers. To do so, we focused on the simpler derivative **1**, the structure of which was optimized using DFT methods. The optimized geometry, which is close to that found experimentally, was then subjected to Natural Bond Orbital (NBO) analysis which identified a bonding orbital connecting the two central heavy atoms (Figure 3). The parentage of this orbital (66.1% Rh/33.9% Sb) indicates that the bonding pair is polarized toward the Rh atom. The polar nature of this linkage contrast with the almost perfectly covalent Pt–Sb bond found in complexes such as **A**.^7a^ Given the polarization of this Sb–Rh interaction, the bonding situation between the two centers is thus best described by invoking two resonance structures. The first one, **I**, corresponds to a Sb^IV^Rh^II^ complex with a covalent bond connecting those two centers. Resonance structure **II** corresponds to a square pyramidal rhodate (Rh^I^) complex stabilized by a Z-type chlorostibonium (Sb^V^) ligand (Figure 3). The Rh–Cl1 bond is also heavily polarized toward the Cl atom, as supported by an NBO analysis which reveals lp(Cl_trans_) → σ*(Rh–Sb) and lp(Cl_trans_) → s(Rh) donor–acceptor interactions (Figure 3). It follows that a third resonance structure (**III**) can be considered to account for the strong polarization of the Rh–Cl bond. These three resonance structures can be reconciled using the dative formalism shown in **IV**. This representation entails a d^8^ square planar rhodium(I) complex whose properties are altered by donation from the filled dz^2^ orbital to the stibonium Z-type ligand. As previously explained,^4,14^ this donation renders the site trans to the Z-type ligands more Lewis acidic, allowing for the coordination of a chloride ligand.

**Figure 3:**
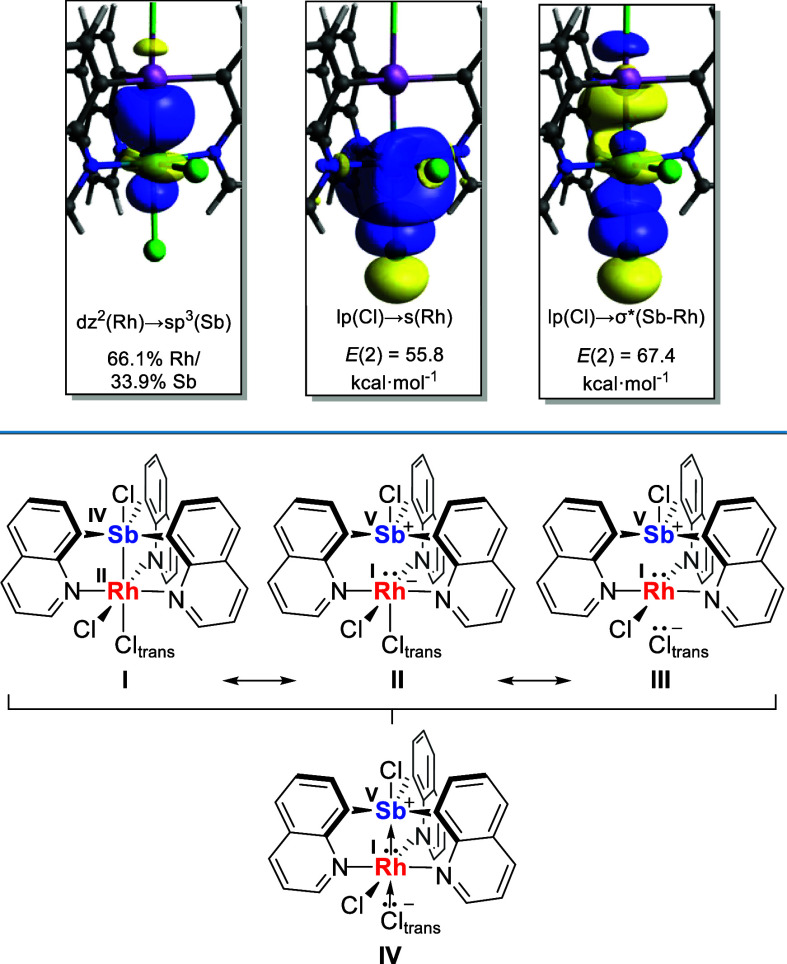
Top: NBOs (isovalue = 0.05) corresponding to the Rh–Sb bond (left), and representative NBOs involved in donor–acceptor interactions between Cl1 and the Rh center (middle and right) with associated *E*(2) values. Bottom: resonance structures of **1** and representation according to the dative formalism.

In short, we have synthesized antimony-centered tripodal ligands adorned with quinolyl donor ligands. These ligands complex rhodium trichloride and, in the process, undergo oxidative insertion of the antimony center in one of the Rh–Cl bonds. The resulting complexes feature a polar Rh–Sb interaction, suggesting an extreme bonding description in which a Rh^I^ center is stabilized by donation to an antimony Z-type ligand. Explorations of these compounds for catalysis are ongoing in our laboratory.

## Supporting Information Available

Additional experimental and computational details (PDF)
Optimized structures in xyz format (XYZ)

## Supplementary Material

om4c00258_si_001.pdf

om4c00258_si_002.xyz

## Notes

We would like to acknowledge an elegant contribution that has just appeared, and that also describes **L_Quin_** as well as its coordination chemistry toward platinum.^15^
