# Supplementary material for: Rh → Sb Interactions Supported by Tris(8-quinolyl)antimony Ligands
Source: Organometallics. 2024 Aug 15;43(17):1785–8. doi: 10.1021/acs.organomet.4c00258 (PMC11389720; doi:10.1021/acs.organomet.4c00258)
Supplement: Supplementary file 1 — om4c00258_si_001.pdf [file om4c00258_si_001.pdf]

Supporting Information

Rh→Sb interactions supported by t*ris*(8-quinolyl)antimony ligands

Casey R. Wade,[1] Brendan L. Murphy,[2] Shantabh Bedajna,[2] and François P. Gabbaï*,[2]

[1]Department of Chemistry & Biochemistry, The Ohio State University, Columbus, Ohio 43210, United States.

[2]François P. Gabbaï – Department of Chemistry, Texas A&M University, College Station, Texas 77843, United States

*Corresponding author email: francois@tamu.edu

Contents

1. Synthesis and characterization

1.1. General considerations S2

1.2. Synthetic procedures & NMR spectra S3

2. Computational details S11

3. References S12

1. Synthesis and characterization

1.1. General considerations

$(MeCN)_3RhCl_3$[1] and 6-methyl-8-bromoquinoline[2] were prepared according to literature procedures. 8-bromoquinoline was purchased from Oakwood and $SbCl_3$ was purchased from Acros; these compounds were used without further purification. THF was dried over Na/K. All other solvents used were ACS reagent grade and used as received. ^1H and ^{13}C spectra were recorded at room temperature on a Bruker Avance 500 spectrometer. Chemical shifts are given in ppm and are referenced to residual ^1H or ^{13}C solvent peaks. Mass spectrometry analysis was performed in-house at the Center for Mass Spectrometry using a Thermo-Scientific Q Exactive Focus instrument. Elemental analysis (EA) was performed at Atlantic Microlab (Norcross, GA).

Crystallographic measurements were performed at 110(1) K using an APEX-II CCD area detector diffractometer (graphite monochromated Mo-Kα radiation, $\lambda = 0.71073$ Å) or a D8 Venture (IμS source Cu-Kα radiation, $\lambda = 1.54178$ Å) diffractometer. In each case, a specimen of suitable size and quality was selected and mounted onto a nylon loop. Integrated intensity information for each reflection was obtained by reducing the data frames using APEX3.[3] The semi-empirical method SADABS was used for the absorption corrections.[4] The structures were solved by direct methods using ShelXT[5] and refined against F^2 with anisotropic temperature-dependent parameters for all non-hydrogen atoms using ShelXL[6] using Olex2.[7] All H-atoms were geometrically placed and refined using a riding model. Diamond4 was used for final data presentation. Positional disorder was found to affect one of the interstitial DMSO molecules in the lattice of **1**. This disorder was modeled, leading to an improvement in the quality of the refinement. CCDC 2362692-2362694 contains the supplementary crystallographic data for this paper. These data can be obtained free of charge via www.ccdc.cam.ac.uk/data_request/cif, or by emailing data_request@ccdc.cam.ac.uk, or by contacting The Cambridge Crystallographic Data Centre, 12 Union Road, Cambridge CB2 1EZ, UK; fax: +44 1223 336033.

1.2. Synthetic procedures

Synthesis of **L_{Quin}**: 8-bromoquinoline (1.00 g, 4.81 mmol) was dissolved in THF (15 mL) and was cooled to -78 °C. A solution of 2.5 M ^{n}BuLi in hexanes (1.9 mL, 4.75 mmol) was then added dropwise to the solution, which then darkened in color to a deep brown and stirred at this temperature for 1 h. A solution of $SbCl_3$ (0.33 g, 1.45 mmol) in THF (5 mL) was then added to that solution dropwise *via* syringe. The resulting solution was allowed to warm to room temp. and stir overnight. Solvent was removed *in vacuo*, and the resulting solid was extracted into CH_2Cl_2 (50 mL) and filtered over Celite. Solvent was removed *in vacuo*, and the resulting residue was triturated in MeOH (~15 mL). The resulting solid was collected by filtration and washed with Et_2O (3 x 15 mL) to yield a yellow powder. Yield: 0.54 g (74%, 1.07 mmol). **^{1}H NMR**: ($CDCl_3$, 500 MHz): δ 8.86-8.85 (m, 3H), 8.18-8.16 (d, J = 8.1 Hz & 1.8 Hz, 3H), 7.77-7.76 (d, J = 8.1 Hz, 3H), 7.38-7.36 (dd, J = 8.3 Hz & 4.3 Hz, 3H), 7.23-7.20 (t, J = 7.7 Hz, 3H), 7.10-7.09 (dd, J = 6.4 Hz, 3H). **^{13}C NMR**: ($CDCl_3$, 128 MHz): δ 152.53 (s), 149.45 (s), 145.13 (s), 138.00 (s), 136.24 (s), 128.09 (s), 127.87 (s), 127.29 (s), 121.02 (s). HRMS-ESI calculated for $C_{27}H_{19}N_3Sb^+$ $[M+H]^+$: 506.0612; found: 506.0605.

Synthesis of **$L_{Quin\text{-}Me}$**: 6-methyl-8-bromoquinoline (1.00 g, 4.50 mmol) was dissolved in THF (30 mL) and was cooled to -78 °C. A solution of 2.5 M ^{n}BuLi in hexanes (2.2 mL, 5.40 mmol) was then added dropwise to the solution, which then turned to a deep red solution and stirred at this temperature for 1 h. A solution of $SbCl_3$ (0.34 g, 1.50 mmol) in THF (10 mL) was then dropwise *via* canula maintaining the same temperature. The resulting solution was allowed to warm to room temperature and stir overnight. The solution was filtered over Celite and further purified *via* flash chromatography over silica gel using hexanes:ethyl acetate (5:1, v/v). The product was obtained as off-white powder. Yield: 0.38 g (46%, 0.5 mmol). **^{1}H NMR**: ($CDCl_3$, 500 MHz): δ 8.78-8.77 (dd, J = 4.0 Hz & 1.3 Hz, 3H), 8.08-8.06 (dd, J = 8.3 Hz & 0.9 Hz, 3H), 7.52 (s, 3H), 7.33-7.31 (dd, J = 8.4 Hz & 4.2 Hz, 3H), 6.94 (broad s, 3H), 2.17 (s, 9H). **^{13}C NMR**: ($CDCl_3$, 128 MHz): δ 151.24 (s), 148.50 (s), 144.54 (s), 140.17 (s), 136.80 (s), 135.47 (s), 128.00 (s), 126.89 (s), 120.89 (s), 21.69 (s). HRMS-ESI calculated for $C_{30}H_{25}N_3Sb^+$ $[M+H]^+$: 548.1081; found: 548.1073.

Synthesis of **1**: $(MeCN)_3RhCl_3$ (33 mg, 9.9 x 10^{-2} mmol) and L_{Quin} (50 mg, 9.9 x 10^{-2} mmol) were suspended in DMSO (1 mL). The solution was heated to reflux, during which time the solids dissolved into solution and the solution turned orange. Upon cooling, a bright yellow microcrystalline solid precipitated, and was suspended in CH_2Cl_2 (~0.5 mL) and collected by filtration. The solid was then washed with CH_2Cl_2 (2 mL) and Et_2O (2 mL) to yield a yellow microcrystalline solid. Single crystals suitable for X-ray diffractometry were produced using this method, which identified the solid as having two interstitial DMSO molecules in the lattice. The yield is calculated to account for those DMSO molecules. Yield: 52 mg (61%, 6.0 x 10^{-2} mmol). Due to poor solubility, satisfactory NMR spectra could not be obtained. Purity is verified by combustion analysis. Elemental analysis calculated for $C_{31}H_{30}N_3O_2S_2Cl_3RhSb$: C 42.71, H 3.47; found C 42.22, H 3.37.

Synthesis of **2**: In a scintillation vial, $(MeCN)_3RhCl_3$ (12 mg, 3.6 x 10^{-2} mmol) and $L_{Quin\text{-}Me}$ (20 mg, 3.6 x 10^{-2} mmol) were suspended in DMSO (3 mL). The solution was heated to reflux, during which the solids dissolved into solution and the solution turned orange. Upon cooling, an orange

suspension formed. A yellow powder was separated from the supernatant upon centrifugation. The supernatant was decanted, and the powder was washed with Et_2O (3 x 2 mL). The pale yellow powder was sparingly soluble in $CDCl_3$. Single crystals of **2**-(H_2O)-2($CHCl_3$) suitable for X-ray diffractometry were produced by layering a $CDCl_3$ solution of the compound with *o*-difluorobenzene. Yield: 11 mg (40%, 1.5 x 10^{-2} mmol). **^{1}H NMR**: ($CDCl_3$, 500 MHz): δ 11.91 (broad s, 1H, rear quinolyl C*H*), 10.78 (broad s, 2H, flanking quinolyl C*H*), 9.26 (d, 2H, *J* = 1.3 Hz, flanking quinolyl C*H*), 9.08 (broad s, 1H, rear quinolyl C*H*), 8.15-8.12 (dd, 2H, *J* = 1.3 Hz & 8.3 Hz, flanking quinolyl C*H*), 8.08-8.06 (dd, 2H, *J* = 0.8 Hz & 8.3 Hz, rear quinolyl C*H*), 7.65 (broad s, 3H, rear quinolyl C*H* & flanking quinolyl C*H*), 7.59 (s, 1H, rear quinolyl C*H*), 7.35-7.32 (m, 2H, flanking quinolyl C*H*), 2.66 (s, 6H, flanking quinolyl CH_3), 2.48 (s, 3H, rear quinolyl CH_3). Due to poor solubility, a satisfactory ^{13}C NMR spectrum could not be obtained. Elemental analysis calculated for $C_{30.95}H_{24.95}Cl_{5.85}N_3RhSb$: C 42.68, H 2.89; found C 42.70, H 3.26. This EA result corresponds to **2**-0.95($CHCl_3$), suggesting partial loss of the interstitial solvents.

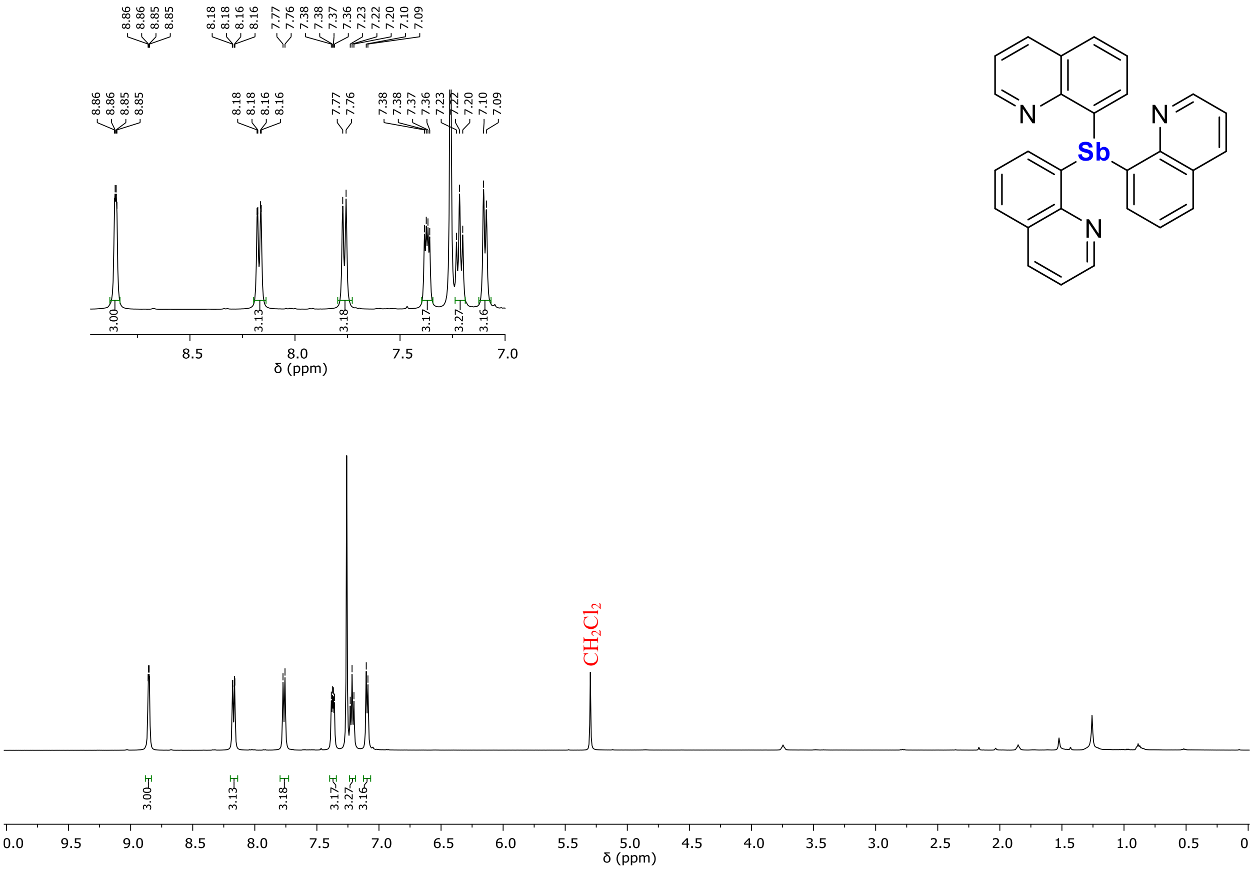

Figure S1. ^{1}H NMR spectrum ($CDCl_3$, 500 MHz) of $\mathbf{L_{Quin}}$.

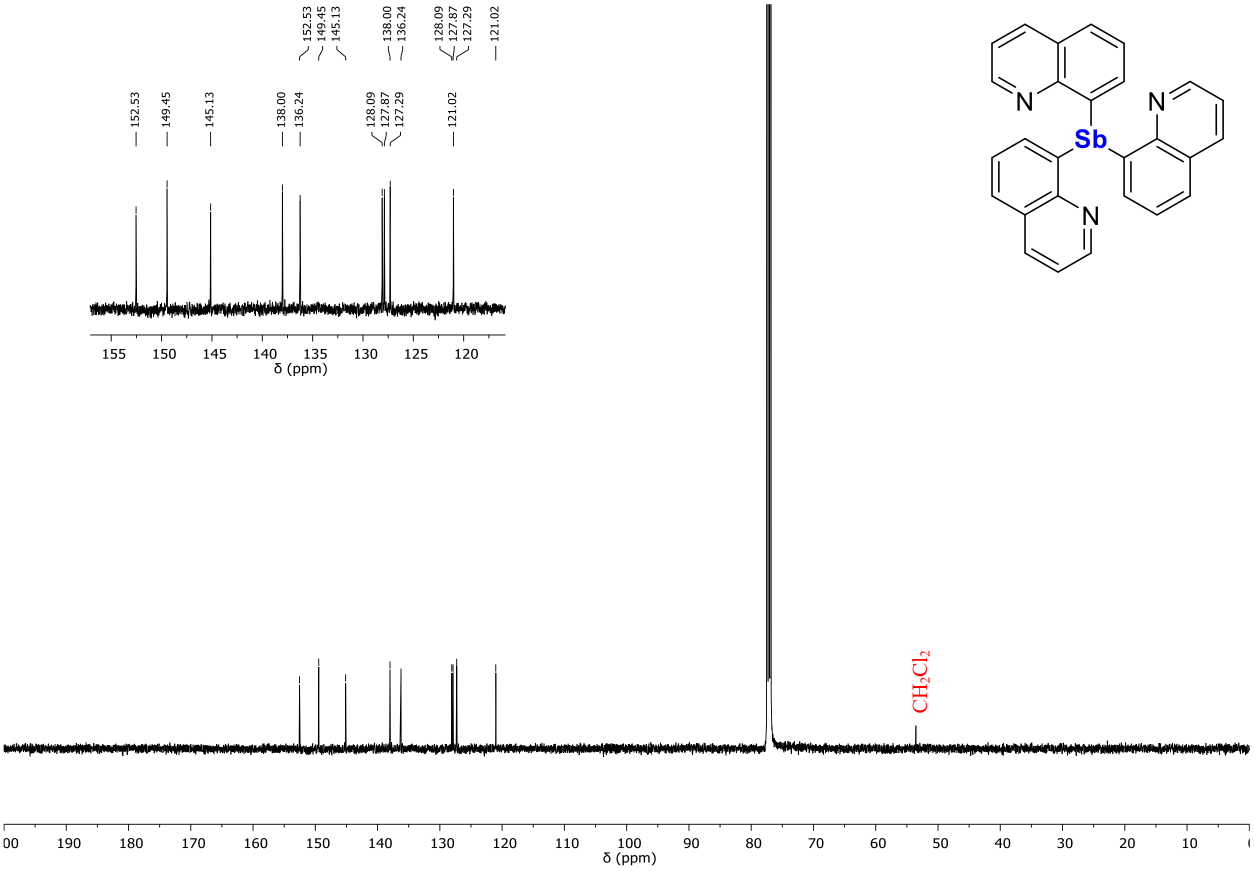

Figure S2. ^{13}C NMR spectrum ($CDCl_3$, 126 MHz) of $\mathbf{L_{Quin}}$. Solvent peak is truncated for clarity.

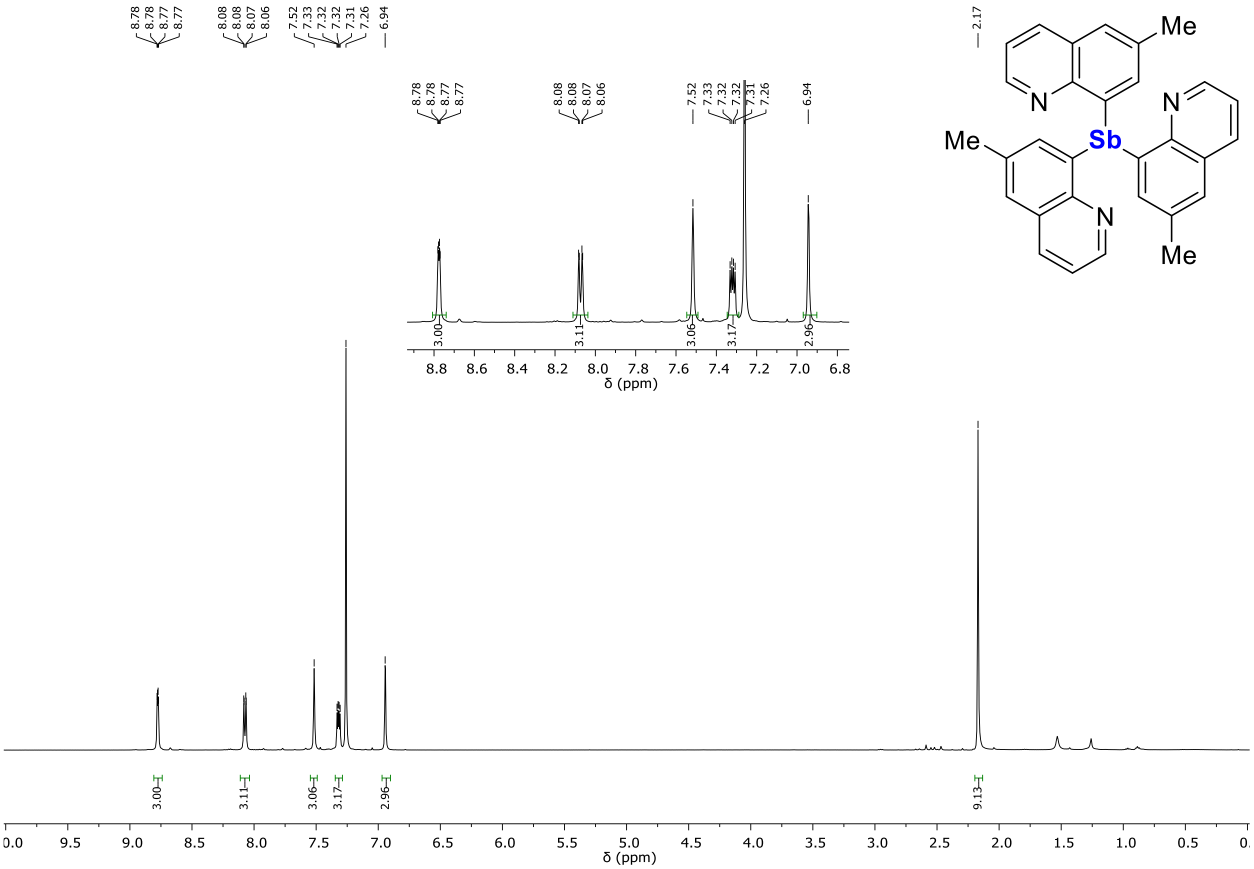

Figure S3. ^{1}H NMR spectrum ($CDCl_3$, 500 MHz) of $\mathbf{L_{Quin\text{-}Me}}$.

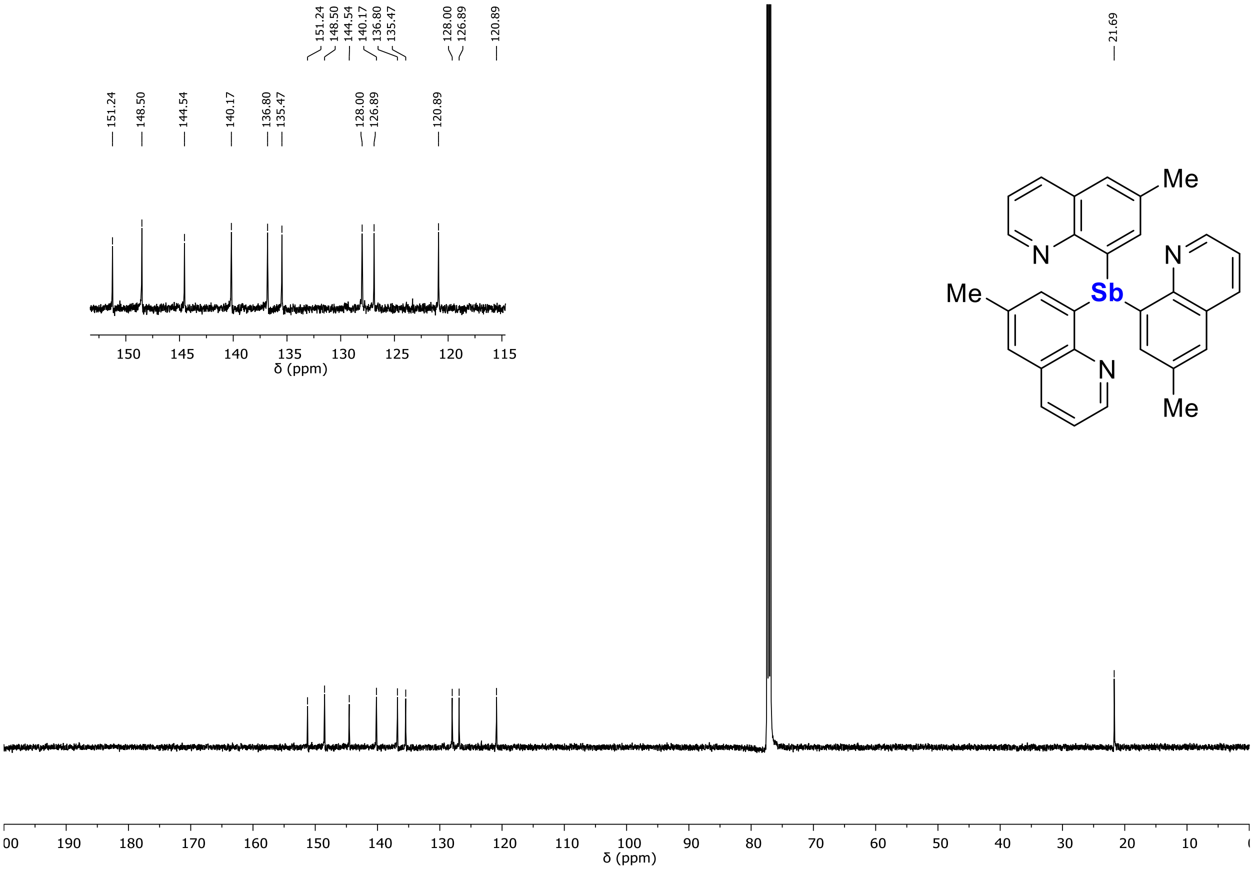

Figure S4. ^{13}C NMR spectrum ($CDCl_3$, 126 MHz) of $\mathbf{L_{Quin\text{-}Me}}$. Solvent peak is truncated for clarity.

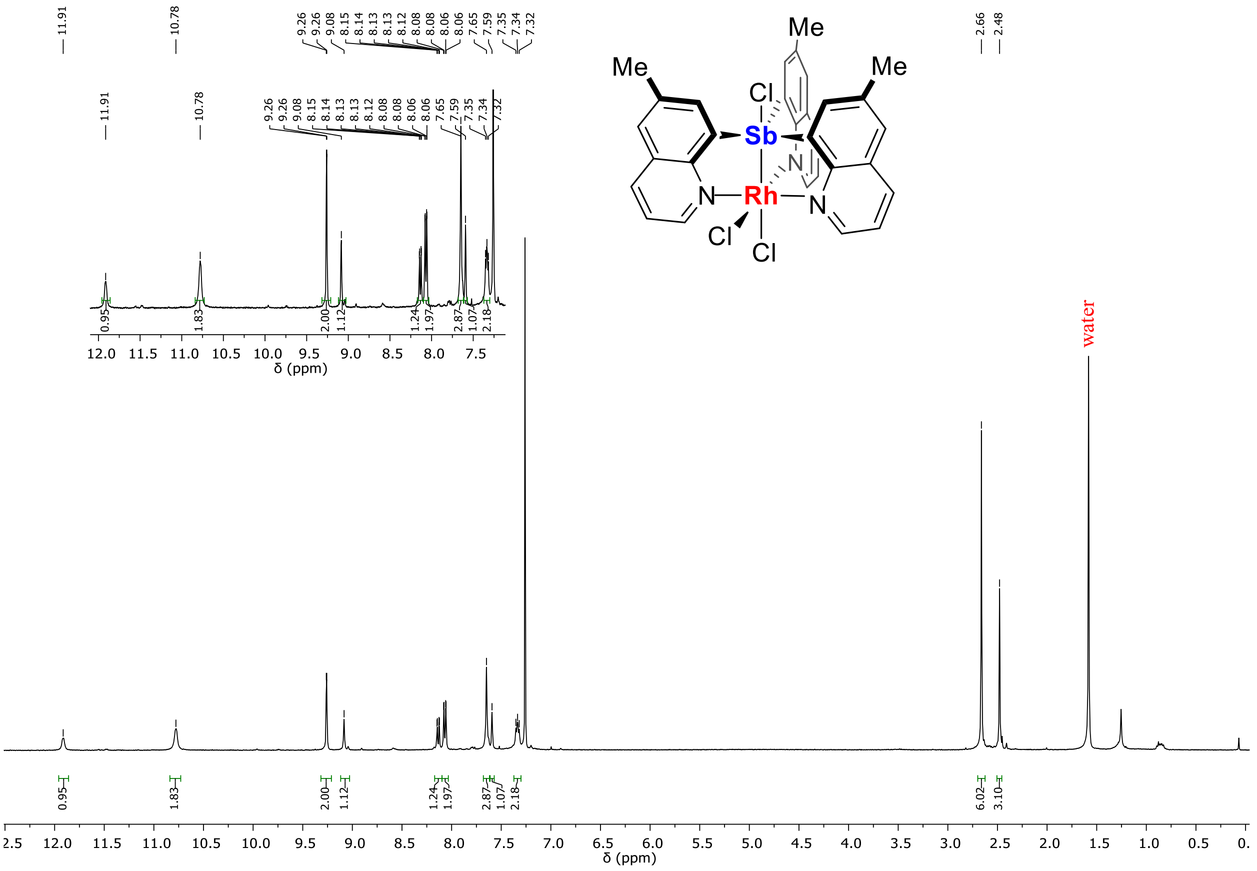

Figure S5. ^{1}H NMR spectrum ($CDCl_3$, 500 MHz) of **2**.

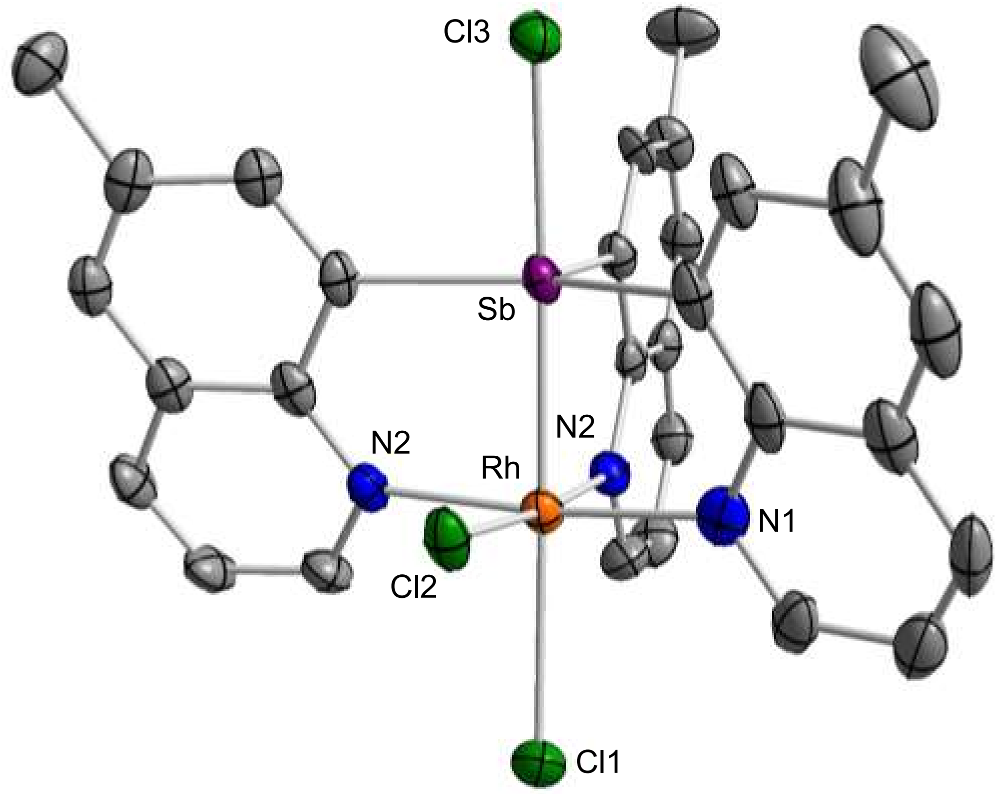

Figure S6. Solid-state structure of **2**. Hydrogen atoms, and interstitial chloroform & water molecules are omitted for clarity. Pertinent metrical parameters can be found in the main text. Color code: purple (Sb), orange (Rh), green (Cl), blue (N), gray (C).

2. Computational details

Density functional theory (DFT) structural optimizations were performed with the Gaussian 16 program.[8] The solid-state geometry of **1** was optimized using the B3LYP functional[9] and the following mixed basis sets: aug-cc-pVTZ-PP[10] for Sb, cc-pVTZ-PP[11] for Rh, 6-311G(d)[12] for Cl, & 6-31G[13] for C, N, and H. For all optimized structures, frequency calculations were performed in order to confirm the absence of imaginary frequencies. NBO calculations were performed with NBO 7.0[14] at the same level of theory and were visualized using Avogadro.[15]

3. References

1. Catsikis, B.; Good, M. L., The preparation and identification of 1,2,3-$RhCl_3(CH_3CN)_3$. *Inorg. Nucl. Chem. Lett.* **1968**, *4*, 529-531.
2. Lee, C.-I.; Zhou, J.; Ozerov, O. V., Catalytic Dehydrogenative Borylation of Terminal Alkynes by a SiNN Pincer Complex of Iridium. *J. Am. Chem. Soc.* **2013**, *135*, 3560-3566.
3. Bruker, 2019, APEX3 (v2019.2011-2010), Bruker AXS Inc., Madison, Wisconsin, USA.
4. Sheldrick, G. M. *SADABS, Version 2007/4*, Bruker Analytical X-ray Systems Inc.: Madison, Wisconsin, USA, 2007.
5. Sheldrick, G. M., SHELXT - integrated space-group and crystal-structure determination. *Acta Crystallogr. A* **2015**, *71*, 3-8.
6. Sheldrick, G. M. *SHELXL-2014: Program for Crystal Structure Refinement*, University of Göttingen, Germany, 2014.
7. Dolomanov, O. V.; Bourhis, L. J.; Gildea, R. J.; Howard, J. A. K.; Puschmann, H., OLEX2: a complete structure solution, refinement and analysis program. *J. Appl. Crystallogr.* **2009**, *42*, 339-341.
8. Frisch, M. J.; Trucks, G. W.; Schlegel, H. B.; Scuseria, G. E.; Robb, M. A.; Cheeseman, J. R.; Scalmani, G.; Barone, V.; Petersson, G. A.; Nakatsuji, H.; Li, X.; Caricato, M.; Marenich, A. V.; Bloino, J.; Janesko, B. G.; Gomperts, R.; Mennucci, B.; Hratchian, H. P.; Ortiz, J. V.; Izmaylov, A. F.; Sonnenberg, J. L.; Williams; Ding, F.; Lipparini, F.; Egidi, F.; Goings, J.; Peng, B.; Petrone, A.; Henderson, T.; Ranasinghe, D.; Zakrzewski, V. G.; Gao, J.; Rega, N.; Zheng, G.; Liang, W.; Hada, M.; Ehara, M.; Toyota, K.; Fukuda, R.; Hasegawa, J.; Ishida, M.; Nakajima, T.; Honda, Y.; Kitao, O.; Nakai, H.; Vreven, T.; Throssell, K.; Montgomery Jr., J. A.; Peralta, J. E.; Ogliaro, F.; Bearpark, M. J.; Heyd, J. J.; Brothers, E. N.; Kudin, K. N.; Staroverov, V. N.; Keith, T. A.; Kobayashi, R.; Normand, J.; Raghavachari, K.; Rendell, A. P.; Burant, J. C.; Iyengar, S. S.; Tomasi, J.; Cossi, M.; Millam, J. M.; Klene, M.; Adamo, C.; Cammi, R.; Ochterski, J. W.; Martin, R. L.; Morokuma, K.; Farkas, O.; Foresman, J. B.; Fox, D. J. *Gaussian 16 Rev. C.01*, Wallingford, CT, 2016.
9. (a) Becke, A. D., Density-functional thermochemistry. III. The role of exact exchange. *J. Chem. Phys.* **1993**, *98*, 5648-5652; (b) Lee, C. T.; Yang, W. T.; Parr, R. G., Development of the Colle-Salvetti Correlation-Energy Formula into a Functional of the Electron-Density. *Phys. Rev. B* **1988**, *37*, 785-789.
10. (a) Peterson, K. A., Systematically convergent basis sets with relativistic pseudopotentials. I. Correlation consistent basis sets for the post-d group 13--15 elements. *J. Chem. Phys.* **2003**, *119*, 11099-11112; (b) Peterson, K. A.; Figgen, D.; Goll, E.; Stoll, H.; Dolg, M., Systematically convergent basis sets with relativistic pseudopotentials. II. Small-core pseudopotentials and correlation consistent basis sets for the post-d group 16--18 elements. *J. Chem. Phys.* **2003**, *119*, 11113-11123; (c) Peterson, K. A.; Shepler, B. C.; Figgen, D.; Stoll, H., On the Spectroscopic and Thermochemical Properties of ClO, BrO, IO, and Their Anions. *J. Phys Chem. A* **2006**, *110*, 13877-13883.
11. Peterson, K. A.; Figgen, D.; Dolg, M.; Stoll, H., Energy-consistent relativistic pseudopotentials and correlation consistent basis sets for the 4d elements Y--Pd. *J. Chem. Phys.* **2007**, *126*, 124101-12.
12. (a) Petersson, G. A.; Bennett, A.; Tensfeldt, T. G.; Al-Laham, M. A.; Shirley, W. A.; Mantzaris, J., A complete basis set model chemistry. I. The total energies of closed-shell atoms and hydrides of the first-row elements. *J. Chem. Phys.* **1988**, *89*, 2193-2218; (b) Petersson, G. A.; Al-Laham, M. A., A complete basis set model chemistry. II. Open-shell systems and the total energies of the first-row atoms. *J. Chem. Phys.* **1991**, *94*, 6081-6090.
13. (a) Ditchfield, R.; Hehre, W. J.; Pople, J. A., Self-Consistent Molecular-Orbital Methods. IX. An Extended Gaussian-Type Basis for Molecular-Orbital Studies of Organic Molecules. *J. Chem. Phys.* **1971**, *54*, 724-728; (b) Hehre, W. J.; Ditchfield, R.; Pople, J. A., Self-Consistent Molecular Orbital Methods. XII. Further Extensions of Gaussian-Type Basis Sets for Use in Molecular Orbital Studies of Organic Molecules. *J. Chem. Phys.* **1972**, *56*, 2257-2261.
14. Glendening, E.D.; Badenhoop, J. K.; Reed, A. E.; Carpenter, J. E.; Bohmann, J. A.; Morales, C. M.; Karafiloglou, P.; Landis, C. R.; Weinhold, F. *NBO 7.0, Theoretical Chemistry Institute*, University of Wisconsin, Madison, WI, 2018.

15. Hanwell, M. D.; Curtis, D. E.; Lonie, D. C.; Vandermeersch, T.; Zurek, E.; Hutchison, G. R., Avogadro: an advanced semantic chemical editor, visualization, and analysis platform. *J. Cheminformatics* **2012**, *4*, 17.
